# Spatial segregation between wild ungulates and livestock outside protected areas in the lowlands of Nepal

**DOI:** 10.1371/journal.pone.0263122

**Published:** 2022-01-27

**Authors:** Shivish Bhandari, Ramiro D. Crego, Jared A. Stabach

**Affiliations:** 1 Natural Science Society, Kathmandu, Nepal; 2 Smithsonian National Zoo and Conservation Biology Institute, Conservation Ecology Center, Front Royal, VA, United States of America; University of Southern Queensland, AUSTRALIA

## Abstract

Understanding how wildlife interacts with human activities across non-protected areas are critical for conservation. This is especially true for ungulates that inhabit human-dominated landscapes outside the protected area system in Nepal, where wildlife often coexists with livestock. Here we investigated how elevation, agricultural land, distance from roads, and the relative abundance of livestock (goats, sheep, cow and buffalo) influenced wild ungulate chital (*Axis axis*), nilgai (*Boselaphustrago camelus*), wild boar (*Sus scrofa*) and sambar (*Rusa unicolor*) abundance and occurrence. We counted all individuals of wild ungulates and livestock along 35 transects conducted between November 2017 and March 2018 in community forests of Bara and Rautahat distracts in the lowlands of Nepal. We assessed abundance and occurrence relation to covariates using Generalized Linear Models. We found that livestock outnumbered wild ungulates 6.6 to 1. Wild boar was the most abundant wild ungulate, followed by nilgai, chital, and sambar. Elevation and livestock abundance were the most important covariates affecting the overall abundance of wild ungulates and the distribution of each individual ungulate species. Our results suggest spatial segregation between wild ungulates, which occur mainly on high grounds (> 300 m.a.s.l.), and livestock that concentrate across low ground habitats (< 300 m.a.s.l.). Our results provide a critical first step to inform conservation in community forest areas of Nepal, where wildlife interacts with people and their livestock. Finding better strategies to allow the coexistence of ungulates with people and their livestock is imperative if they are to persist into the future.

## Introduction

The global environmental crisis is pushing a myriad of species to the brink of extinction [1–3]. Among the most vulnerable species are large terrestrial mammals [4–6] particularly herbivores [7], which have experienced sharp population declines due to the ongoing and massive anthropogenic pressure on terrestrial ecosystems. Over the next 50 years, the global human population is expected to exceed 10 billion [8, 9], putting increasing pressure on ecosystems. Formal protected areas play a vital role in conserving biodiversity [10, 11], however, only a few are large enough to encompass the ecological and territorial needs to sustain large mammal populations [5, 12]. As a result, most terrestrial large mammal migrations are in sharp decline or already extinct [13, 14]. Moreover, protected areas tend to concentrate human population density at their edges [10, 15], restricting animal mobility and leading to increased human-wildlife conflict, including increased incidences of poaching, and competition and/or predation of livestock [4, 10, 16–18]. Finding solutions that ensure the coexistence of wildlife with humans, especially across areas with no form of environmental protection, is crucial for the future of conservation of these species [5, 17].

In developing countries, rural poverty tends to increase the demand for access to natural resources [19–21], with forests being among the most impacted of all ecosystems. Forest loss has been particularly pervasive in Asia. Supporting ~9% (about 700 million) of the global human population [22, 23], Asia has experienced high deforestation rates (> 300 km^2^ /year; [24]). Most forests are cleared to increase the size of pastures for increasing densities of livestock [19, 25] resulting in consequential large-scale declines in local biodiversity [6, 26–29]. Currently, one-quarter of all Asian mammal species are now threatened with extinction and in urgent need of improved conservation strategies [30], including ungulate species such as pygmy hog (*Porcula salvania*), Indian mouse deer (*Moschiola indica*), swamp deer (*Cervus duvaucelii*), gaur (*Bos gaurus*), and four-horned antelope (*Tetracerus quadricornis*) [31]. To protect large mammals, conserving large tracts of mature forests across human-dominated landscapes is crucial [19, 32].

Nepal is home to a wide diversity of habitats, from tropical (~60 m. above sea level (m.a.s.l.)) to high-mountain (> (up to 8849m.a.s.l.) systems, supporting an incredible array of species [33]. Formal protection for these habitats, however, is severely limited, with only 23.3% of the land surface currently under any form of protection [33]. Over half of all ungulate species are threatened with extinction [33, 34]. Approximately 29% of the forested land in Nepal (areal estimate >16,000 km^2^) is managed under community forestry practices by local and state entities [33, 35]. The annual rate of forest loss across these areas was 0.9%, or approximately 28 km^2^ per year [35–37]. Human population is also on the rise, leading to concomitant increases in livestock. This has raised conservation concerns as livestock can spatially displace wild ungulates, forcing wildlife to forage in low-quality food areas via exploitative and interference competition, and leading to reduced fitness [38, 39]. Habitat loss can also force wild ungulatesto seek for foraging opportunities in agricultural areas, increasing crop damage and leading to conflict with farmers [40, 41]. This conflict has, for instance, decreased population abundance of many ungulates species across Nepal [41].

Understanding the relationship between ungulates and the environment can contribute to the sustainable management of these species, items that are critical for the conservation of Nepal’s megafauna [38, 42, 43]. To date, comparatively little attention has been paid to how anthropogenic pressure, including farmers and their livestock, drive the distribution and habitat selection of ungulates outside Nepalese protected areas. In this study, we investigated how elevation, agricultural land, distance from roads, and the relative abundance of livestock ((cow (*Bostaurus indicus*), buffalo (*Bubalus arnee*), goats (*Capra hircus*), and sheep (*Ovis aries*)) influenced the abundance and occurrence of wild ungulates (chital (*Axis axis*), nilgai (*Boselaphustrago camelus*), wild boar (*Sus scrofa*) and sambar (*Rusa unicolor*)), with the goal of providing information to help design more sustainable practices to ensure the coexistence of wild species and people that are dependent on these ecosystems for survival.

## Materials and methods

### Ethics

The proposal was approved by the Committee on Department of Forestry, Ministry of Forest and Environment, Kathmandu, Nepal. The research permission number was #990-2072-2073. We performed this research under the Forest Act 2049 and 2051, and the National Parks and Conservation Act 2029 and 2030 of Government of Nepal.

### Study area

**Fig 1:**
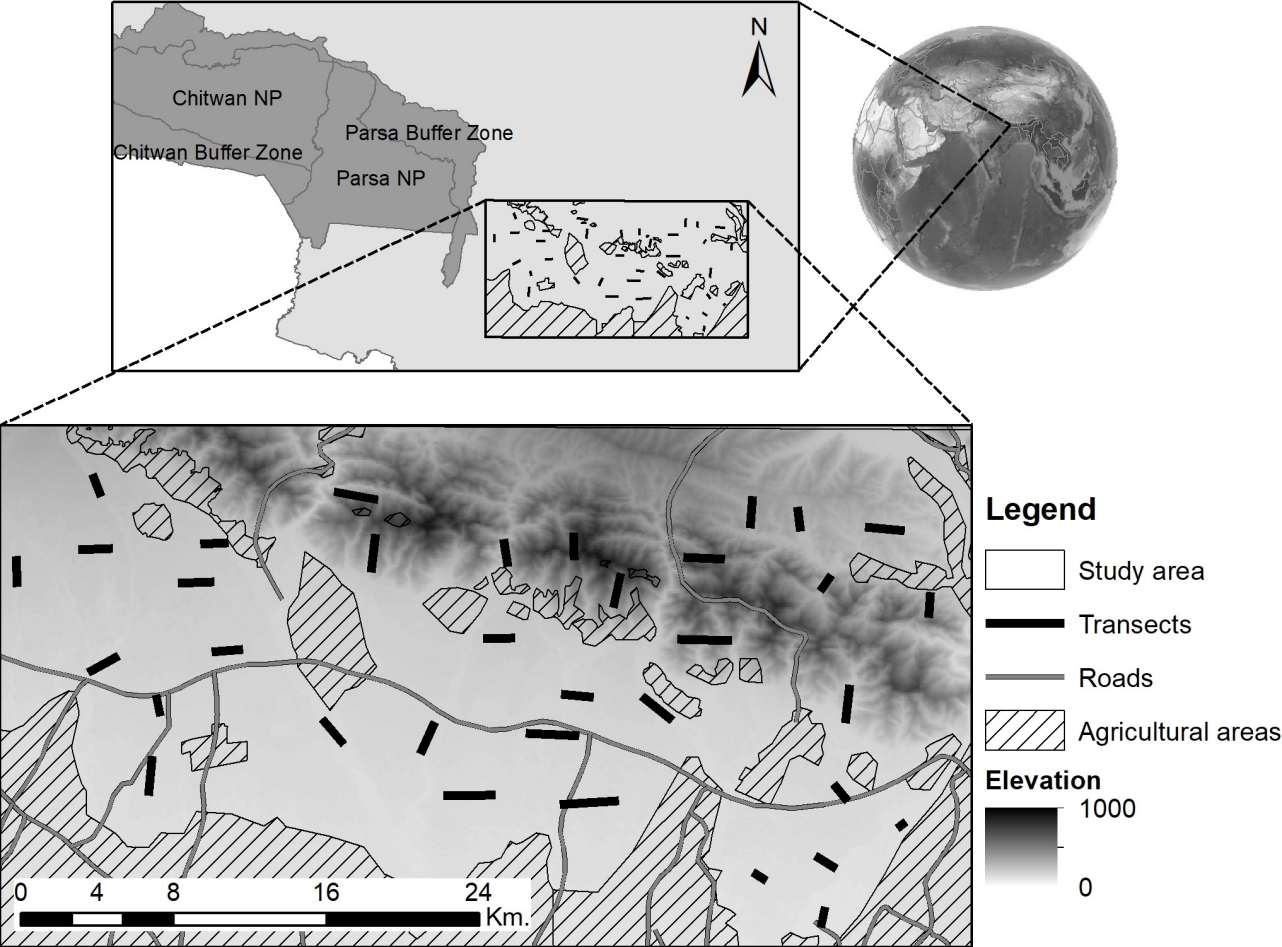
Study site locations (forests in Bara and Rautahat) in the lowland of Nepal.

This study was conducted across an approximate 300 km^2^ region in the community forests of Bara and Rautahat districts in the lowlands of central-eastern Nepal (26.9–27.4° N; 84.9–85.2° E) (Fig 1). The terrain is dominated by tropical forests, with an elevation range of 80 to 800 m.a.s.l. The landscape is covered by tropical forest, dominated by Sal (*Shorea robusta*) and acacia (*Acacia catechu*) species. The area borders Parsa National Park to the west, the Mahabharat mountains to the North, agricultural lands and human settlements to the south, and other community forest and scattered human settlement to the east (Fig 1). Most of the local people in this area rely on agriculture and livestock farming for subsistence. The main crops are corn (*Zea mays*), wheat (*Triticum aestivum*), potato (*Solanum tuberosum*), and rice (*Oryza sativa*). Main livestock species include cow (*Bostaurus indicus*), buffalo, goats, and sheep. Forest products, harvested for subsistence, include firewood, leaves, and wood.

The community forests in the Bara and Rautahat are home to more than fifty mammalian species including large predators (e.g., tiger (*Panthera tigris*), common leopard (*Panthera pardus*), and striped hyenas (*Hyaena hyaena*)), mega herbivores (e.g., Elephants (*Elephas maximus*), one-horned rhinoceros (*Rhinoceros unicornis*)), and large herbivores (e.g., chital, nilgai, wild boar and sambar) [44, 45]. In this study, we focused specifically on the effects on chital, nilgai, wild boar, and sambar. Importantly, our study site is one of the major corridors for elephants and a core habitat range in Nepal for tiger [42, 45, 46].

### Data collection

Between November 2017 and March 2018, we conducted 35 line transects to count the number of livestock and wild ungulate species. Starting point of each transect was selected randomly across animal trails, roads or riverbeds that are accessible by foot. Transects were then walked by two observers in a straight line following a random angle on the compass. Because of terrain difficulties, transects length varied (mean transect length = 1648 m ± 496 SD; range = 573 to 2799 m). Transects were spaced > 2 km apart to maintain independence (Fig 1). All aforementioned wild and domestic animals present within 100 m from the center of the transect were counted. We focused on chital, nilgai, wild boar and sambar as they were the only large species detected and per their body sizes we could assume that detectability was not biasing results.

We incorporated a set of covariates that we thought *a priori* could affect the abundance and occurrence of these ungulate species. The study area presents a marked north-south elevation change. Thus, we included mean elevation for each transect, derived from a 90-m digital elevation model [47]. To account for the effect of roads on species occurrence, we obtained road information from the Nepalese Department of Survey (Kathmandu, Nepal), given the recognized importance of roads in adversely affecting ungulate occurrence across the region [48]. To account for the potential effect of agricultural areas, we manually digitized all agricultural patches identified using high resolution Google Earth imagery (Google Inc., Mountain View, CA, USA) using QGIS 3.12.1 [49]. We estimated the mean Euclidean distance of each transect to the nearest river, road or agricultural area (S1 Fig). We used the raster package in R for all geospatial analyses [50].

To account for the potential effect of livestock on wild ungulates [51], we calculated the total number of individuals of all four species of livestock counted along the transect and divided this value by the length of the transect (ind/km), providing a transect-level measure of relative abundance.

### Statistical analysis

We used generalized linear models (GLMs) with a Poisson distribution to investigate the factors explaining ungulate abundance across the landscape. We used an offset (log of transect length) to account for different sampling efforts due to different transect lengths [52]. We modeled ungulate abundance in relation to distance to roads, distance to agricultural fields, elevation, and livestock relative abundance. For this analysis, we added the counts of all ungulates to evaluate how the community responds to each of the different covariates.

Additionally, we investigated single species responses to the mentioned covariates. The relatively small sample sizes and high variation in counts for each species impeded us from investigating single species abundance responses. Instead, we transformed the response variable into presence/absence to investigate single species occurrence probability in relation to the different covariates, using a GLM with binomial distribution and *logit* function [52, 53].

Before fitting models, we checked that none of the continuous covariates presented collinearity by calculating Pearson r correlations (< 0.7) and conducting a Variance Inflation Factor analysis (VIF < 3). We standardized all variables to a mean of zero and one unit of standard deviation for analysis. For each modeling procedure, we fit all model combinations with all covariates. We used Akaike’s Information Criterion corrected for small sample sizes (AICc) to perform model selection [53]. We selected the most parsimonious models based on a ΔAICc< 2 and model averaged results to calculate parameter estimates and 95% confidence intervals (CI) [53], using the MuMIn and *AICcmodavg* packages in R [50].

## Results

Among wild species, wild boars were the most abundant (49.7%), followed by nilgai (27.3%), chital (20.0%), and sambar (3.03%; Table 1). The most abundant livestock type was cow (54.9%), followed by goats (24.9%), buffalo (17.0%), and sheep (3.0%; Table 1). Livestock relative abundance was 6.6 times higher than all wild species combined (livestock = 20.71 ind/km vs wild species = 3.14 ind/km).

**Table 1:** Wild and domestic species animal counts and prevalence at 35 transects across the study area in Nepal.

	Total number of animals	Mean ind/km (+-SE)	Prevalence (%)
Wild species			
Chital	33	0.59 (0.21)	34.29
Wild boar	82	1.54 (0.45)	42.86
Sambar	5	0.12 (0.06)	11.43
Nilgai	45	0.90 (0.37)	28.57
Total	165	3.14 (0.87)	54.28
Livestock			
Goat	271	5.30 (2.23)	42.86
Sheep	33	0.91 (0.66)	20.00
Cow	597	10.48 (3.66)	65.71
Buffalo	185	4.02 (2.06)	11.43
Total	1086	20.71 (6.00)	74.29

### Ungulate ensemble abundances

**Fig 2:**
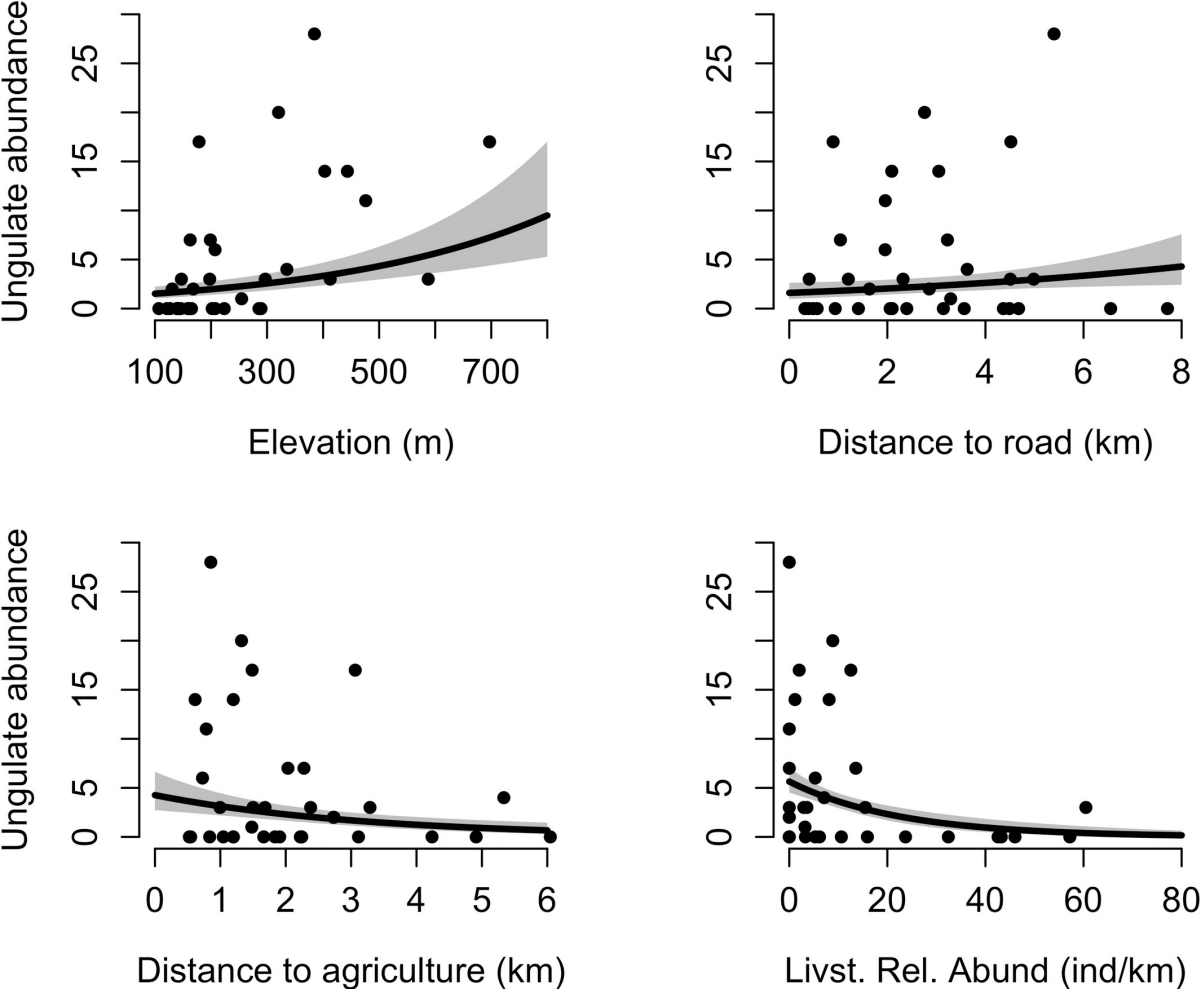
Ungulate assemblage abundance prediction (95% confidence intervals) from the most parsimonious models in relation to elevation (m), distance to roads (km), distance to agriculture (km), and livestock relative abundance (Livst. Rel. Abund) in Nepal.

The most parsimonious models explaining ungulate assemblage abundance accounted for 77% of cumulative weight and included all four covariates (elevation, distance to roads, distance to agriculture, and livestock relative abundance (S1 Table)). Model results indicated that the ungulate assemblage abundance increased with increasing elevation (0.37 [95% CI: 0.23–0.51]) and increasing distance from roads (0.23 [95% CI: 0.03–0.42]) but decreased with increasing distance from agricultural areas (-0.42 [95% CI: -0.65 –-0.19]) and increasing relative livestock abundance (-1.60 [95% CI: -2.26 –-0.93]) (Fig 2). All variables were significant (confidence intervals did not overlap 0).

### Ungulate single-species occurrence

The six most parsimonious models that best explained chital presence accounted for 93% cumulative model weight and included the four covariates (elevation, distance to roads, distance to agriculture and livestock relative abundance; Tables 2 and S2). We found two models that best explained the presence of wild boar, which together accounted for 60% of the model weight and included three covariates (elevation, distance to roads, and livestock relative abundance; Table 2). Four models best explained sambar’s presence and accounted for 55% model weight. For this species, however, the null model was the most parsimonious model, suggesting a lack of explanatory power (Table 2). This is due to the small number of detections (*n* = 5) for this species (Table 1). For nilgai, three models best explained its presence probability, which accounted for 49% of the model weight and included elevation and livestock relative abundance (Table 2).

**Table 2:** Model selection results to investigate five species of ungulate occurrence in Nepal.

Species	Model	K	AICc	ΔAICc	W	Cum. W
Chital	Elev + Dist. Road	3	29.85	0.00	0.20	0.20
	Elev + Dist. Road + Dist. Agr	4	29.95	0.10	0.19	0.39
	Elev + Dist. Road + Dist. Agr. + Liv. Abund.	5	30.31	0.46	0.16	0.54
	Elev + Liv. Abund.	3	30.60	0.75	0.14	0.68
	Elev + Dist. Road + Liv. Abund.	4	30.68	0.83	0.13	0.81
	Elev + Dist. Agr. + Liv. Abund.	4	30.85	1.00	0.12	0.93
Wild boar	Elev + Liv. Abund.	3	38.45	0.00	0.42	0.42
	Elev + Dist. Road + Liv. Abund.	4	40.26	1.81	0.17	0.59
Sambar	Null model	1	27.00	0.00	0.19	0.19
	Elev	2	27.30	0.30	0.17	0.36
	Liv. Abund.	2	28.12	1.13	0.11	0.47
	Dist. Agr	2	28.76	1.76	0.08	0.55
Nilgai	Elev	2	42.47	0.00	0.26	0.26
	Null model	1	44.00	1.53	0.12	0.38
	Elev + Liv. Abund.	3	44.15	1.68	0.11	0.49

Only the most parsimonious models are presented, i.e. ΔAICc< 2. The explanatory variables are elevation (m), distance to road (km), distance to agricultural fields (km), and livestock relative abundance. K = number of estimated parameters; AICc = Akaike’s Information Criterion corrected for small samples; ΔAICc = differences in AICc, W = model weight, and Cum. W. = cumulative model weight.

**Fig 3:**
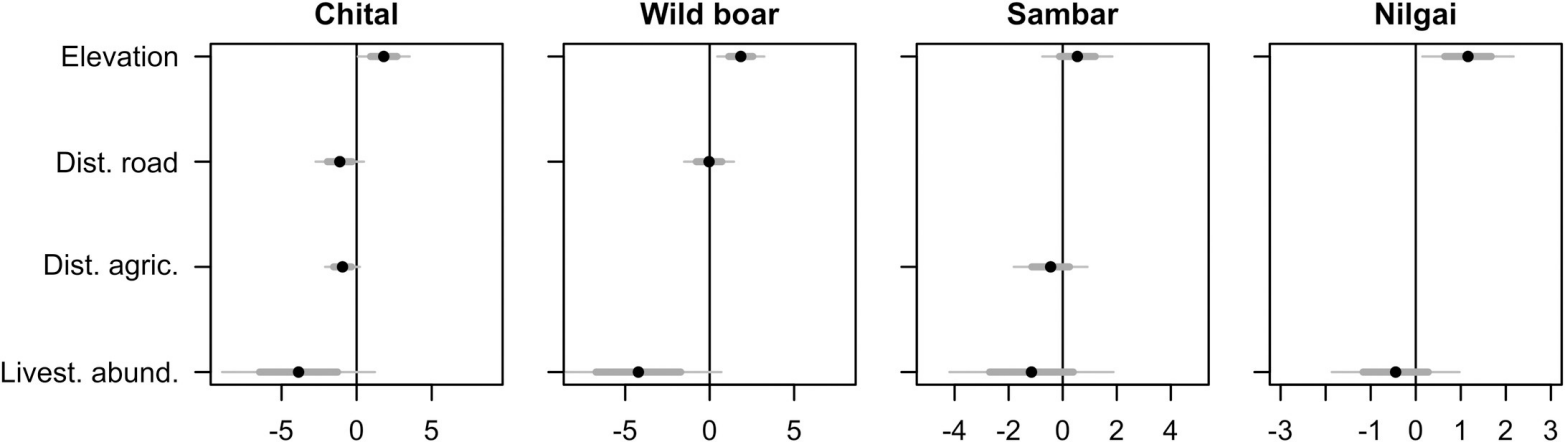
Average parameter responses from the most parsimonious models for occurrence probability of five species of ungulates in Nepal. Models for each species include a different number of parameters. Black dots indicate parameter means, thin grey lines indicate 95% confidence intervals, and thick grey lines indicate unconditional standard errors.

**Fig 4:**
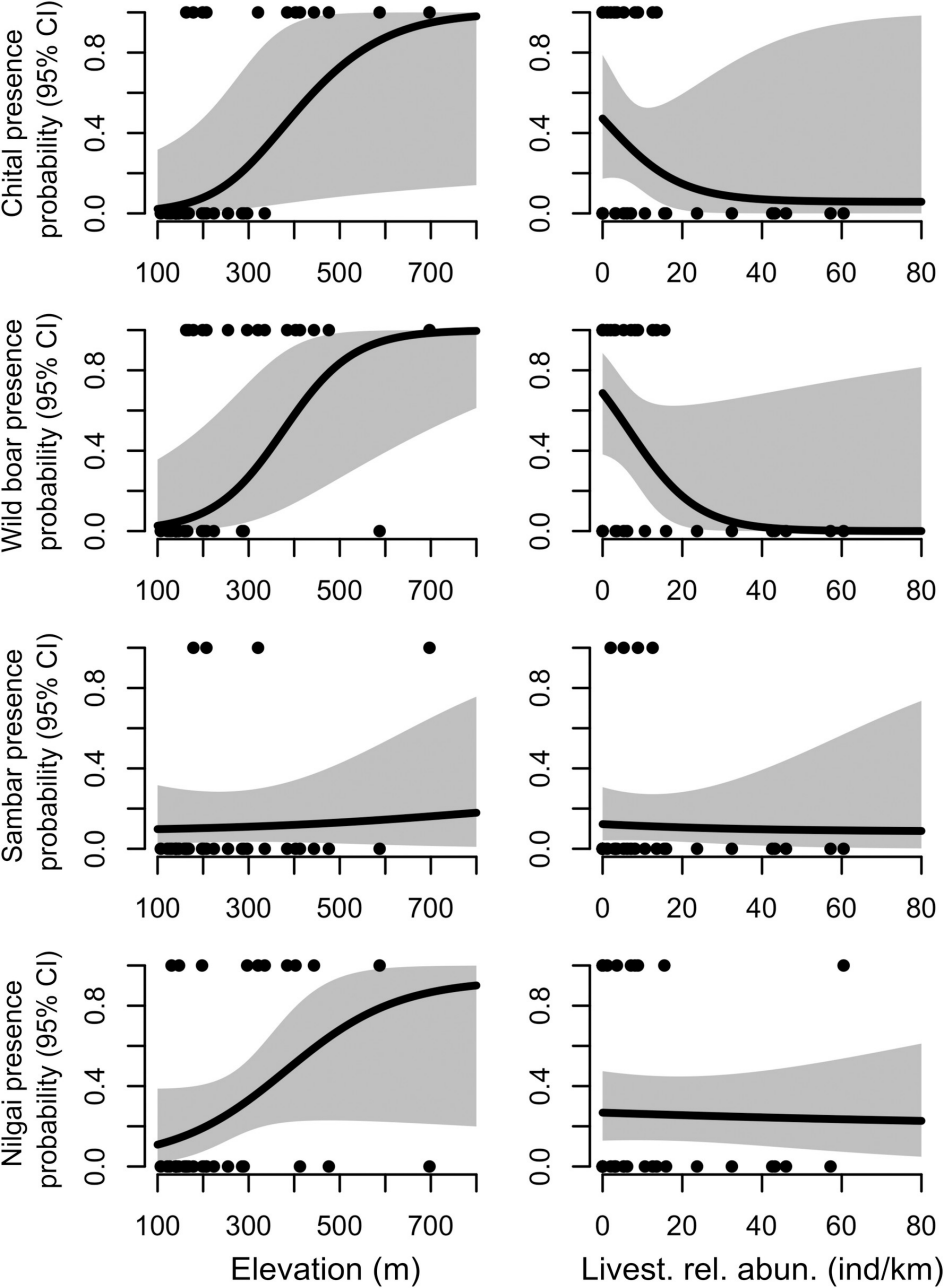
Averaged parameter predictions from the most parsimonious models for occurrence probability of wild ungulates in Nepal in relation to elevation (m) and relative livestock abundance (ind/km).

Elevation was the most important covariate explaining the presence probability of all ungulates, with presence probability increasing with increasing elevation (Figs 3 and 4). Distance to the nearest road was negatively related to the presence of chital, positively related to the presence of wild boar (Fig 3). Chital presence was slightly negatively associated with the distance to the nearest agricultural area, whereas sambar presence probability varied little with changes in distance to the nearest agricultural area (Fig 3). Finally, ungulates’ presence probability was negatively associated with increasing livestock relative abundance (Fig 3). This relationship was much stronger for chital and wild boar, but with little change for the other two species (Fig 4). In all these trends, 95% confidence intervals overlapped zero.

## Discussion

In this study, we found livestock abundance outnumbered wild species abundance by 6.6:1 in the community managed forests of Nepal. The overall abundance of the wild ungulate species was negatively related to livestock abundance and positively related to elevation. Similarly, all five species of wild ungulates occurred mainly in elevated areas (> 300 m.a.s.l.) dominated by Sal forests, mixed type forest (forests mostly dominated by *Terminalia alata*, *Adina cordifolia*, *Schimawallichii* and *Dalbergia sissoo*), and overall fewer human disturbances in comparison to the lowland forests. Results suggest that livestock, which occur predominantly on low grounds (93% of livestock < 300 m.a.s.l.), are excluding wild herbivores, which in turn occur mainly on high grounds (69% of ungulates > 300 m.a.s.l.). More data are certainly needed to identify significant effects on large-herbivore occurrence at the single species level. However, the effect of livestock on the community abundance and the trends we report on single species are largely informative for a system that is rapidly losing forest cover and that needs urgent action to protect critical habitat [54, 55].

The chital is one of the most abundant deer species in Nepal [56, 57]. However, the distribution of this species is almost entirely restricted to protected areas [43, 56]. Our finding of 0.59 individual/km is significantly lower than reported for Nepal’s protected areas. While our estimates are not directly comparable, previous research has reported abundances that are clearly much higher than our study area (84.7 ± 7.9 ind/km2, [43]; 31.73±4.26 ind/km2, [56]. We found that chital preferred elevated areas and areas with lower livestock abundance. Livestock higher abundance and anthropogenic activities in the lower elevated areas (< 300 m.a.s.l.) might explain chital preference towards elevated areas. Our result supported those from [43, 58], who found that chital are using suboptimal habitats given high pressure from human related activities.

The wild boar is one of the most widely distributed species in Nepal, occurring from lowland (< 1000m.a.s.l) to the mid-hills (around 2500 m.a.s.l) [57, 59, 60]. Thus, our findings that wild boar was the most abundant wild species is not surprising. We also found that wild boar preferred elevated areas and avoided livestock and roads, similarly to findings by [40]. Many studies identified the wild boar as one of the major drivers of human-wildlife conflict in protected areas of Nepal [59]. However, based on our field observations (unpublished data), we did not find any crop damage by the wild boar. Furthermore, our results did not show a relationship between wild boar locations and agricultural land. Our results suggest that wild boars occur only in areas with low human impact, likely reinforced by hunting pressure [40, 59].

The distribution of sambar in Nepal is mostly restricted to Parsa, Chitwan, Banke, and Bardia National Parks, and includes nearby habitats. The abundance and density of the sambar in the central lowland, such as Chitwan National Park and Bardia National Park, are comparatively higher than estimates from eastern (this study) and western Nepal (Suklaphanta National Park: [33]. Sambars are highly sensitive to anthropogenic pressures [61–63]. Consequently, we found sambar to be the rarest species in our study (0.12 mean individual/km). In most parts of the country, this species prefers floodplains with grass and riverine forest [43, 63]. The lack of floodplains with abundant grass in the study area could also explain low sambar abundance.

Nilgai is endemic to the Indian subcontinent [64]. Nepal’s lowland region represents a small population (< 400 individual) of nilgai which is sparsely distributed, mostly outside of Nepal’s protected areas [41, 65, 66]. We found nilgai to be the second most abundant species of our species surveyed. Its presence was positively associated with higher elevation and negatively associated with increasing livestock abundance. This species may be competing with livestock or alternatively, conflict with local farming communities because of crop damage may be pushing the species into the highlands where human impact is lower [41, 66]. With most nilgai populations occurring outside protected areas, regulating human activities to ensure that wild ungulates can coexist with livestock will be critical for the future of this species.

### Implication for conservation

Wildlife conservation outside the protected area system of Nepal is challenging. Increases in human population are exacerbating pressures on natural resources, with concomitant increases in deforestation and habitat fragmentation [32, 35, 41]. Results from our study suggest that high livestock abundance in the lowland forests are excluding ungulates, which occur mainly in elevated areas where livestock abundance is much lower. Conditions of these elevated areas, however, may be less favorable to sustain high population abundances and can compromise the population stability of these species. The high abundance of livestock in non-protected areas of Nepal may compromise the future presence of wild ungulates [67].

More research is certainly needed to confirm the trends found in this study, as our limited sample size and lack of seasonal variation in the data makes it hard to generalize results on a wide scale. Moreover, previous research has shown that in grassland ecosystems ungulates and livestock coexistence is possible [54, 55, 68, 69]. It may be important to focus research on better understanding the livestock species specific stocking rates that benefit producers without compromising the future of wild ungulate populations in forest ecosystems. This can provide critical information to manage and, regulate the abundance of livestock in this human dominated landscape for the conservation of wild ungulate species.

Additionally, declining wild ungulates in the natural environment can be the cause of human-carnivore conflict, with predators focusing on domesticated prey that have replaced wild species [70, 71]. This might not only affect ungulate distributions, but also alter prey-predator relationships [71, 72]. Most of the community forests outside the protected areas systems have to be managed properly. Because our study site is connected to the Parsa National Park, it serves as an important biological corridor and potential habitat for many large predators. The conservation of wild ungulates is important to maintain natural predator-prey relationships, as well as to minimize human-wildlife conflict [38, 43, 44].

In addition, protected landscapes in lowland Nepal have not been successful in supporting populations of many species in sharp decline, such as nilgai [41, 66] and sambar [43]. These species are known to be a major portion of the diet of charismatic species such as tiger [43]. Extending protection outside formal protected areas boundaries, while also incorporating ecotourism opportunities, can be beneficial to the community in order to generate alternative income sources with minimum human-wildlife conflict. Management approaches with dual goals of regulating livestock grazing and improving habitat conditions for wild ungulates and other species, would be helpful for sustainable biodiversity conservation in the lowlands of Nepal.

## Conclusion

In this study, we documented that high livestock abundances in the non-protected communal forests of Nepal are highly affecting the abundance and presence of many wild ungulates. The high livestock abundance found in the lowland forests appears to be explaining the presence of wild ungulates mainly on higher lands, thus, ungulates are being extirpated from the lowlands. These findings are important, not only for the large herbivores described here, but also for other critical endangered species that depend on these habitats for survival. With formal reserves across Nepal being insufficient to protect the space use needs of many large terrestrial animals, management strategies that favor the coexistence of wild ungulates with human activities is imperative for the future of wildlife.

## Supporting information

S1 Fig The study site location: Distance of each line transect to elevation, road and agricultural area. (DOCX)

S1 Table Model selection results to investigate ungulate assemblage abundances in relation to elevation (m), distance to road (km), distance to agricultural fields (km) and livestock relative abundance (ind/km), in Nepal. K = number of estimated parameters; AICc = Akaike’s Information Criterion corrected for small samples; ΔAICc = difference in AICc, W = model weight, and Cum. W. = cumulative model weight.
(DOCX)

S2 Table Model selection results to investigate five species of ungulate occurrence in Nepal. The explanatory variables are elevation (m), distance to road (km), distance to agricultural fields (km), and livestock relative abundance. K = number of estimated parameters; AICc = Akaike’s Information Criterion corrected for small samples; ΔAICc = differences in AICc, W = model weight, and Cum. W. = cumulative model weight.
(DOCX)
