# Supplementary material for: Spatial segregation between wild ungulates and livestock outside protected areas in the lowlands of Nepal
Source: PLoS One. 2022 Jan 27;17(1):e0263122. doi: 10.1371/journal.pone.0263122 (PMC8794147; doi:10.1371/journal.pone.0263122)
Supplement: S1 Fig — (DOCX) [file pone.0263122.s001.docx]

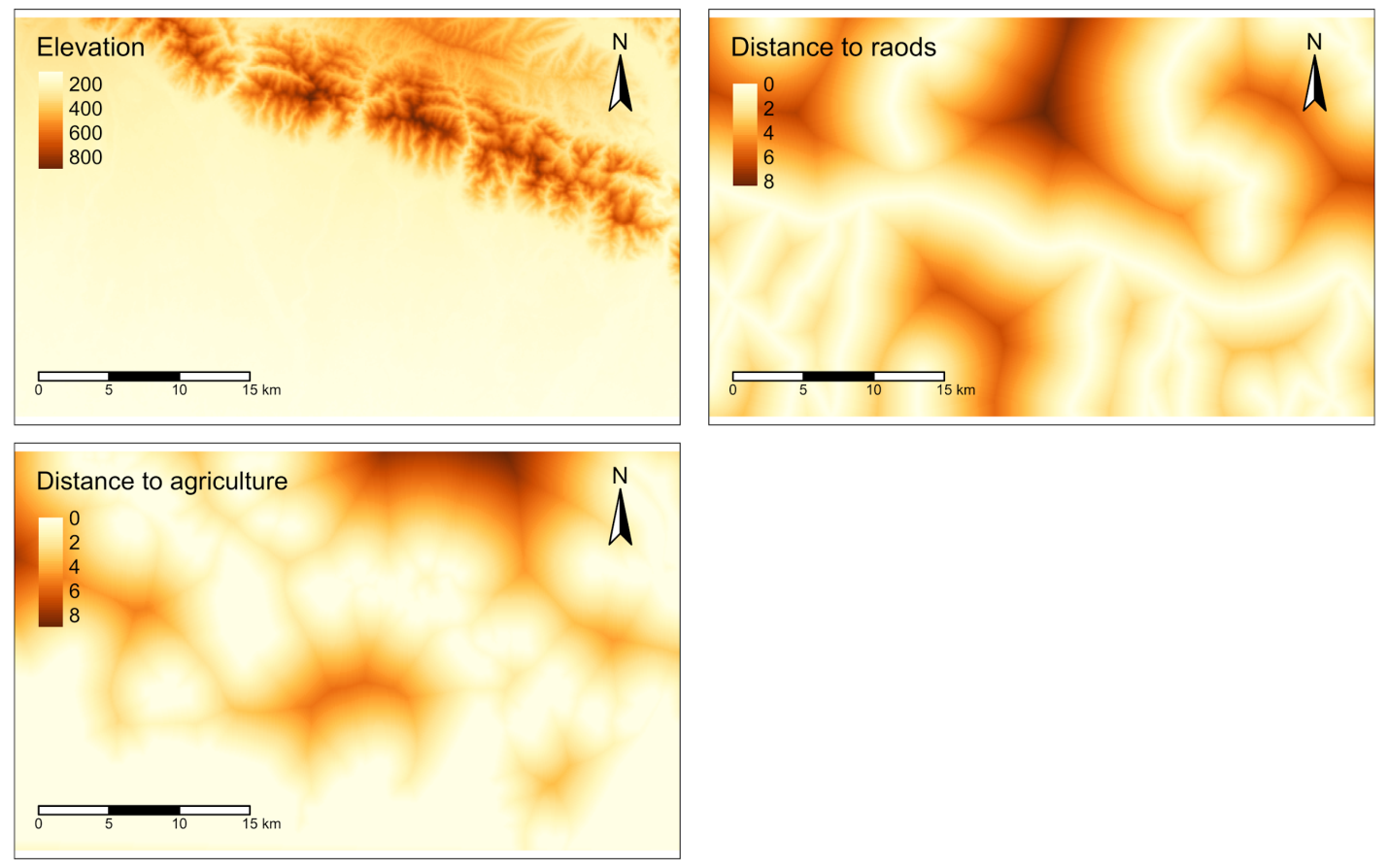


**S1 Fig.** The study site location: distance of each line transect to elevation, road and agricultural area.
