# Supplementary material for: Spatial segregation between wild ungulates and livestock outside protected areas in the lowlands of Nepal
Source: PLoS One. 2022 Jan 27;17(1):e0263122. doi: 10.1371/journal.pone.0263122 (PMC8794147; doi:10.1371/journal.pone.0263122)
Supplement: S1 Table — K = number of estimated parameters; AICc = Akaike’s Information Criterion corrected for small samples; ΔAICc = difference in AICc, W = model weight, and Cum. W. = cumulative model weight. (DOCX) [file pone.0263122.s002.docx]

**S1 Table.** Model selection results to investigate ungulate assemblage abundances in relation to elevation (m), distance to road (km), distance to agricultural fields (km) and livestock relative abundance (ind/km), in Nepal. K = number of estimated parameters; AICc = Akaike’s Information Criterion corrected for small samples; ΔAICc = difference in AICc, W = model weight, and Cum. W. = cumulative model weight.

| **Model** | **K** | **AICc** | **ΔAICc** | **W** | **Cum. W** |
| --- | --- | --- | --- | --- | --- |
| Elev + Dist. Road + Dist. Agr. + Liv. Abund. | 5 | 282.69 | 0.00 | 0.77 | 0.77 |
| Elev + Dist. Agr. + Liv. Abund. | 4 | 285.08 | 2.39 | 0.23 | 1.00 |
| Elev + Dist. Road + Liv. Abund. | 4 | 295.41 | 12.72 | 0.00 | 1.00 |
| Elev + Liv. Abund. | 3 | 301.33 | 18.64 | 0.00 | 1.00 |
| Dist. Road + Dist. Agr. + Liv. Abund. | 4 | 304.42 | 21.73 | 0.00 | 1.00 |
| Dist. Agr. + Liv. Abund. | 3 | 315.35 | 32.66 | 0.00 | 1.00 |
| Elev + Dist. Agr | 3 | 317.40 | 34.71 | 0.00 | 1.00 |
| Dist. Road + Dist. Agr. + Elev | 4 | 319.95 | 37.26 | 0.00 | 1.00 |
| Elev | 2 | 331.91 | 49.22 | 0.00 | 1.00 |
| Dist. Road + Liv. Abund. | 3 | 333.13 | 50.44 | 0.00 | 1.00 |
| Dist. Road + Elev | 3 | 334.16 | 51.47 | 0.00 | 1.00 |
| Liv. Abund. | 2 | 355.09 | 72.40 | 0.00 | 1.00 |
| Dist. Agr | 2 | 371.80 | 89.11 | 0.00 | 1.00 |
| Dist. Road + Dist. Agr. | 3 | 372.15 | 89.46 | 0.00 | 1.00 |
| Dist. Road | 2 | 403.12 | 120.43 | 0.00 | 1.00 |
| Null model | 1 | 405.73 | 123.04 | 0.00 | 1.00 |
