# Supplementary material for: Spatial segregation between wild ungulates and livestock outside protected areas in the lowlands of Nepal
Source: PLoS One. 2022 Jan 27;17(1):e0263122. doi: 10.1371/journal.pone.0263122 (PMC8794147; doi:10.1371/journal.pone.0263122)
Supplement: S2 Table — The explanatory variables are elevation (m), distance to road (km), distance to agricultural fields (km), and livestock relative abundance. K = number of estimated parameters; AICc = Akaike’s Information Criterion corrected for small samples; ΔAICc = differences in AICc, W = model weight, and Cum. W. = cumulative model weight. (DOCX) [file pone.0263122.s003.docx]

**S2 Table.** Model selection results to investigate five species of ungulate occurrence in Nepal. The explanatory variables are elevation (m), distance to road (km), distance to agricultural fields (km), and livestock relative abundance. K = number of estimated parameters; AICc = Akaike’s Information Criterion corrected for small samples; ΔAICc = differences in AICc, W = model weight, and Cum. W. = cumulative model weight.

| **Species** | **Model** | **K** | **AICc** | **ΔAICc** | **W** | **Cum. W** |
| --- | --- | --- | --- | --- | --- | --- |
| Chital | Elev + Dist. Road | 3 | 29.85 | 0.00 | 0.20 | 0.20 |
|  | Elev + Dist. Road + Dist. Agr | 4 | 29.95 | 0.10 | 0.19 | 0.39 |
|  | Elev + Dist. Road + Dist. Agr. + Liv. Abund. | 5 | 30.31 | 0.46 | 0.16 | 0.54 |
|  | Elev + Liv. Abund. | 3 | 30.60 | 0.75 | 0.14 | 0.68 |
|  | Elev + Dist. Road + Liv. Abund. | 4 | 30.68 | 0.83 | 0.13 | 0.81 |
|  | Elev + Dist. Agr. + Liv. Abund. | 4 | 30.85 | 1.00 | 0.12 | 0.93 |
|  | Elev | 2 | 32.98 | 3.12 | 0.04 | 0.97 |
|  | Elev + Dist. Agr. | 3 | 33.73 | 3.87 | 0.03 | 1.00 |
|  | Dist. Agr. + Liv. Abund. | 3 | 39.75 | 9.90 | 0.00 | 1.00 |
|  | Dist. Road + Dist. Agr. + Liv. Abund. | 4 | 42.09 | 12.24 | 0.00 | 1.00 |
|  | Liv. Abund. | 2 | 42.12 | 12.27 | 0.00 | 1.00 |
|  | Dist. Road + Liv. Abund. | 3 | 43.87 | 14.02 | 0.00 | 1.00 |
|  | Dist. Agr | 2 | 45.84 | 15.99 | 0.00 | 1.00 |
|  | Null model | 1 | 47.13 | 17.27 | 0.00 | 1.00 |
|  | Dist. Road + Dist. Agr. | 3 | 48.16 | 18.31 | 0.00 | 1.00 |
|  | Dist. Road | 2 | 49.36 | 19.51 | 0.00 | 1.00 |
|  |  |  |  |  |  |  |
| Wild boar | Elev + Liv. Abund. | 3 | 38.45 | 0.00 | 0.42 | 0.42 |
|  | Elev + Dist. Road + Liv. Abund. | 4 | 40.26 | 1.81 | 0.17 | 0.59 |
|  | Elev + Dist. Agr. + Liv. Abund. | 4 | 40.97 | 2.52 | 0.12 | 0.71 |
|  | Dist. Road + Liv. Abund. | 3 | 41.91 | 3.47 | 0.07 | 0.78 |
|  | Elev | 2 | 42.45 | 4.00 | 0.06 | 0.84 |
|  | Elev + Dist. Road + Dist. Agr. + Liv. Abund. | 5 | 42.96 | 4.52 | 0.04 | 0.88 |
|  | Liv. Abund. | 2 | 43.29 | 4.85 | 0.04 | 0.92 |
|  | Dist. Road + Dist. Agr. + Liv. Abund. | 4 | 44.23 | 5.78 | 0.02 | 0.94 |
|  | Elev + Dist. Road | 3 | 44.50 | 6.05 | 0.02 | 0.96 |
|  | Elev + Dist. Agr. | 3 | 44.85 | 6.40 | 0.02 | 0.98 |
|  | Dist. Agr. + Liv. Abund. | 3 | 45.13 | 6.69 | 0.01 | 0.99 |
|  | Elev + Dist. Road + Dist. Agr | 4 | 47.05 | 8.61 | 0.01 | 1.00 |
|  | Null model | 1 | 49.92 | 11.48 | 0.00 | 1.00 |
|  | Dist. Road | 2 | 51.76 | 13.31 | 0.00 | 1.00 |
|  | Dist. Agr | 2 | 51.84 | 13.39 | 0.00 | 1.00 |
|  | Dist. Road + Dist. Agr. | 3 | 53.86 | 15.41 | 0.00 | 1.00 |
|  |  |  |  |  |  |  |
| Sambar | Null model | 1 | 27.00 | 0.00 | 0.19 | 0.19 |
|  | Elev | 2 | 27.30 | 0.30 | 0.17 | 0.36 |
|  | Liv. Abund. | 2 | 28.12 | 1.13 | 0.11 | 0.47 |
|  | Dist. Agr | 2 | 28.76 | 1.76 | 0.08 | 0.55 |
|  | Elev + Liv. Abund. | 3 | 29.03 | 2.03 | 0.07 | 0.62 |
|  | Elev + Dist. Road | 3 | 29.17 | 2.17 | 0.07 | 0.69 |
|  | Dist. Road | 2 | 29.17 | 2.17 | 0.07 | 0.76 |
|  | Elev + Dist. Agr. | 3 | 29.48 | 2.49 | 0.06 | 0.81 |
|  | Dist. Agr. + Liv. Abund. | 3 | 29.97 | 2.97 | 0.04 | 0.86 |
|  | Dist. Road + Liv. Abund. | 3 | 30.52 | 3.52 | 0.03 | 0.89 |
|  | Dist. Road + Dist. Agr. | 3 | 31.04 | 4.04 | 0.03 | 0.92 |
|  | Elev + Dist. Road + Liv. Abund. | 4 | 31.34 | 4.34 | 0.02 | 0.94 |
|  | Dist. Road + Dist. Agr. + Liv. Abund. | 4 | 31.35 | 4.35 | 0.02 | 0.96 |
|  | Elev + Dist. Road + Dist. Agr | 4 | 31.47 | 4.47 | 0.02 | 0.98 |
|  | Dist. Road + Dist. Agr. + Liv. Abund. | 4 | 32.51 | 5.51 | 0.01 | 0.99 |
|  | Elev + Dist. Road + Dist. Agr. + Liv. Abund. | 5 | 33.79 | 6.79 | 0.01 | 1.00 |
|  |  |  |  |  |  |  |
| Nilgai | Elev | 2 | 42.47 | 0.00 | 0.26 | 0.26 |
|  | Null model | 1 | 44.00 | 1.53 | 0.12 | 0.38 |
|  | Elev + Liv. Abund. | 3 | 44.15 | 1.68 | 0.11 | 0.49 |
|  | Elev + Dist. Road | 3 | 44.70 | 2.23 | 0.09 | 0.58 |
|  | Liv. Abund. | 2 | 44.72 | 2.25 | 0.08 | 0.66 |
|  | Elev + Dist. Agr. | 3 | 44.78 | 2.31 | 0.08 | 0.75 |
|  | Dist. Road | 2 | 46.21 | 3.74 | 0.04 | 0.79 |
|  | Dist. Agr | 2 | 46.25 | 3.78 | 0.04 | 0.83 |
|  | Elev + Dist. Agr. + Liv. Abund. | 4 | 46.64 | 4.17 | 0.03 | 0.86 |
|  | Elev + Dist. Road + Liv. Abund. | 4 | 46.70 | 4.23 | 0.03 | 0.89 |
|  | Dist. Road + Liv. Abund. | 3 | 46.78 | 4.31 | 0.03 | 0.92 |
|  | Dist. Agr. + Liv. Abund. | 3 | 47.11 | 4.64 | 0.03 | 0.95 |
|  | Elev + Dist. Road + Dist. Agr | 4 | 47.17 | 4.70 | 0.02 | 0.97 |
|  | Dist. Road + Dist. Agr. | 3 | 48.61 | 6.14 | 0.01 | 0.98 |
|  | Dist. Road + Dist. Agr. + Liv. Abund. | 4 | 49.33 | 6.86 | 0.01 | 0.99 |
|  | Elev + Dist. Road + Dist. Agr. + Liv. Abund. | 5 | 49.36 | 6.89 | 0.01 | 1.00 |
|  |  |  |  |  |  |  |
